# Smoking and passive smoking increases mortality through mediation effect of cadmium exposure in the United States

**DOI:** 10.1038/s41598-023-30988-z

**Published:** 2023-03-08

**Authors:** Joon Kim, Hangyul Song, Junghoon Lee, Yoon Jung Kim, Hye Soo Chung, Jae Myung Yu, Gyuho Jang, Raekil Park, Wankyo Chung, Chang-Myung Oh, Shinje Moon

**Affiliations:** 1 Department of Biomedical Science and Engineering, Gwangju Institute of Science and Technology, Gwangju, Korea; 2 Department of Internal Medicine, Hallym University Kangnam Sacred Heart Hospital, Hallym University College of Medicine, Seoul, Korea; 3 Department of Public Health Science, Graduate School of Public Health, Seoul National University, Seoul, Korea

**Keywords:** Diseases, Respiratory tract diseases, Risk factors, Medical research, Epidemiology

## Abstract

Cigarette smoking is one of the leading causes of preventable and premature death worldwide. Even worse, many people are generally exposed to passive smoking, which leads to several respiratory diseases and related mortalities. Considering, more than 7000 compounds are included in cigarettes, their combustion results intoxicants that have deleterious effects on health. However, there is a lack of research analyzing the effects of smoking and passive smoking on all-cause and disease-specific mortality through its chemical compounds including heavy metals. Thus, this study aimed to evaluate the effect of smoking and passive smoking on all-cause and disease-specific mortality mediated by cadmium, one of the representative smoking-related heavy metals using data from the National Health and Nutrition Examination Survey (NHANES) 1999–2018 in the United States. We found that current smoking and passive smoking was related to increased risk of all-cause, CVD-related, and cancer-related mortality. Notably, passive smoking showed a synergistic effect with smoking status on the risk of mortality. In particular, current smokers with passive smoking had the highest risk of all-cause and disease-specific deaths. In addition, the accumulation of cadmium in the blood due to smoking and passive smoking mediates the increased risk of all-cause mortality. Further studies are needed to monitor and treat cadmium toxicity to improve smoking-related mortality rates.

## Introduction

Cigarette smoking is one of the leading causes of preventable and premature death worldwide. According to a 2017 World Health Organization (WHO) report, smoking causes more than 7 million deaths each year. Smoking is related to most fatal diseases, including cardiovascular disease (CVD), stroke, diabetes, chronic obstructive pulmonary disease (COPD), and cancer^1,2^. The proportion of responsibility for smoking-related deaths was one-fourth for CVD and 80% for lung cancer and COPD in U.S. adults, respectively^3^. In studies of smoking cessation, former smoking increased the risk of several types of cancer and CVD compared to never-smokers, even though the risk of former smokers was much lower than that of current smokers^4,5^. Even worse, many people are generally exposed to passive smoking, which increases the risk of morbidity and mortality of lung cancer and CVD^6–11^. According to WHO, in 2017, passive smoking was responsible for 1.5 million deaths from chronic respiratory diseases, 1.2 million deaths from cancer, and 60,000 deaths in children under the age of 5 years from respiratory infections^12^. Passive smoking is also strongly correlated with COPD and other types of cancers, such as brain, sinus, breast, and leukemia^13–16^. Although a number of studies reported the harmful effect of cigarette smoking and passive smoking, little is known about the effect of passive smoking stratified by smoking status on human health including all-cause and disease-specific mortality. When considering that smoker frequently have impaired pulmonary function and comorbidity^1,2^, the effect of passive smoking on vulnerable individuals should be evaluated.

More than 7000 compounds are included in commercially available cigarettes and their combustion results intoxicants that have deleterious effects on health^17^. Chemical components of cigarettes include heavy metals and some persistent organic pollutants (POPs). Those components have been known to cause diseases including CVD, cancer, and COPD^18–20^. In addition, some heavy metals increase all-cause and disease-specific mortality in several diseases, such as cancer and heart disease^21–25^. Especially, cadmium is one of the typical heavy metals associated with smoking. When Cd is absorbed into the body, it enters the bloodstream and circulate via erythrocytes. In addition, it accumulates in kidneys with a half-life of 10–30 years^26^. According to epidemiological research, Cd exposure is linked to renal disease, osteoporosis, factures, and CVD^27–31^. Furthermore, exposure of Cd is linked to a higher death rate. Urinary Cd concentrations were related to all-cause mortality, according to a meta-analysis of nine cohort studies^32^. However, there is a lack of research on the effect of cadmium exposed from cigarette smoke on the risk of mortality and morbidity.

The purpose of this study was to examine the effect of smoking and passive smoking on all-cause and disease-specific mortality using a large population-based study of the US population (National Health and Nutrition Examination Survey [NHANES] study). In addition, we performed a mediation analysis to identify cadmium responsible for smoking-related mortality.

## Materials and methods

### Study population

**Figure 1:**
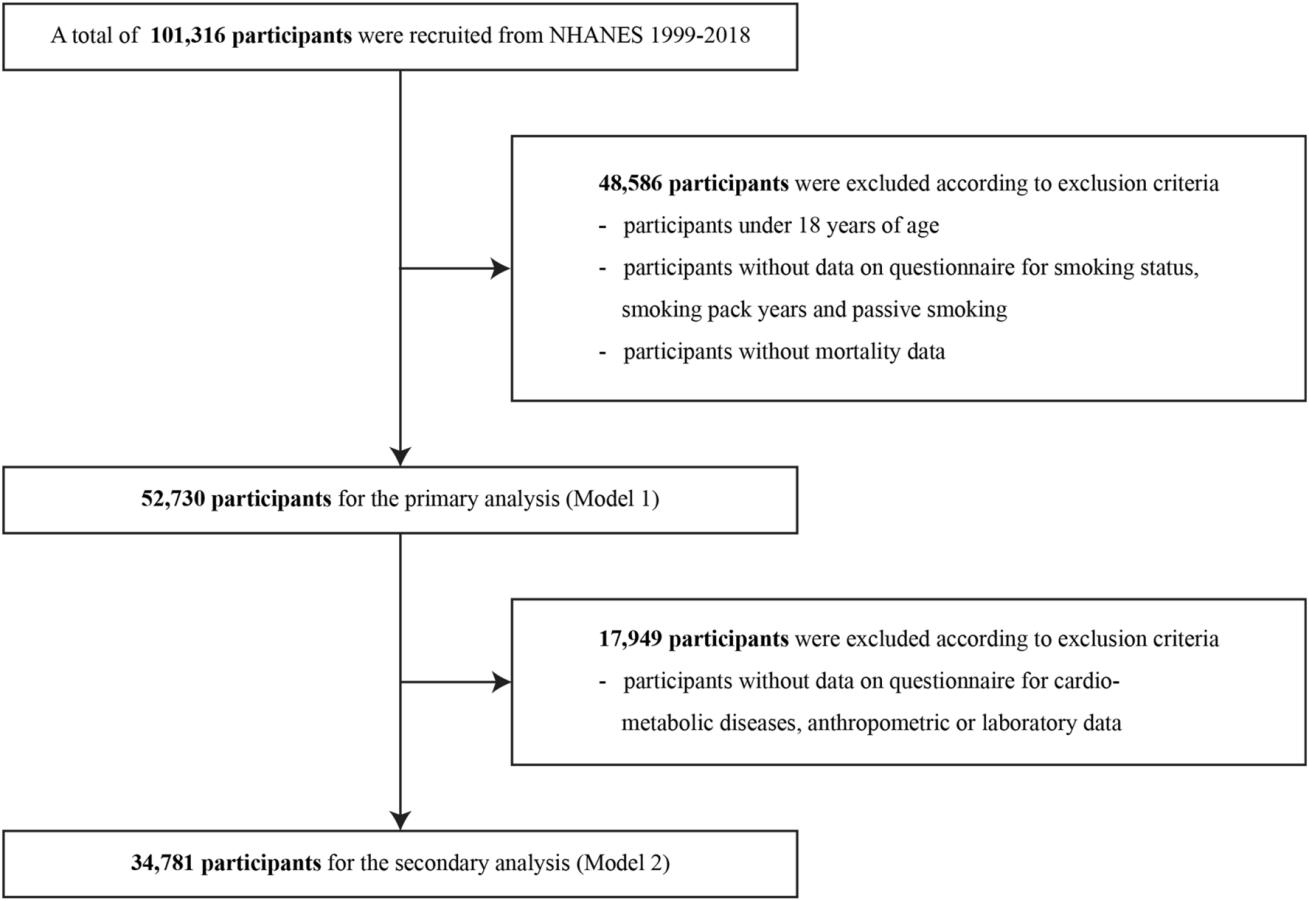
Flowchart for final selection. NHANES, National Health and Nutrition Examination Survey.

The NHANES comprises biennial cross-sectional data including demographic, dietary, and questionnaire data for current health status and past medical history, physical examination data, and laboratory data with a representative sample of the US population. We used a cross-sectional study cohort (NHANES 1999–2018)^33^ and linked the data with the National Death Index to obtain mortality information^34^. Among a total of 101,316, 52,730 participants were included in primary analysis (Model 1) according to the following exclusion criteria: participants aged under 18 years, participants who did not have mortality data, participants who did not respond to the questionnaire for smoking status, smoking pack years, and passive smoking (N = 48,586). Among the participants enrolled in primary analysis (Model 1), 34,781 participants were included in secondary analysis to adjust for comorbidities (Model 2) after excluding 17,949 participants with missing anthropometric data, data for comorbidities, including diabetes mellitus (DM), hypertension, dyslipidemia, and CVD, and laboratory data (Fig. 1).

### Classification of smoking status

Smoking status was investigated using the following structured questionnaire: “*Have you smoked at least 100 cigarettes in your entire life?”; “Do you now smoke cigarettes?”* Passive smoking status was investigated using the following question: “*Does anyone who lives here smoke cigarettes, cigars, or pipes anywhere inside this home?”.*

All participants were categorized according to smoking status, consisting of never-smoker, former smokers, and current smokers, and the presence of passive smoking. We calculated smoking pack-years to estimate cumulative exposure to smoking. In addition, we used the blood cotinine concentration (ng/mL) to estimate the extent of current exposure to tobacco smoke.

### Measurements of covariates

Covariates were age, sex, race/ethnicity, alcohol consumption, and underlying diseases in the baseline survey. The underlying diseases included cancer, COPD, DM, hypertension, dyslipidemia, and CVD events. Data on age, sex, race/ethnicity, and alcohol consumption were acquired from the questionnaire responses. Hypertension was defined as a systolic blood pressure > 140 mmHg, mean diastolic blood pressure > 90 mmHg, or treatment for hypertension. DM was defined as fasting blood glucose > 126 mg/dL, random blood glucose > 200 mg/dL, HbA1c > 6.5%, or treatment for diabetes. Dyslipidemia was defined as fasting total cholesterol level of 240 mg/dL or treatment for dyslipidemia. Cancer history was investigated using structured questionnaires as follows: “*Has a doctor or other health professional ever told you that you had cancer or a malignancy?”, “What kind of cancer was it?”.*

Since 30 types of cancer have been reported, we categorized cancer types into blood, urogenital, gastrointestinal, respiratory, skin, and other cancers for analysis. COPD was investigated using the following questionnaire: “*Has a doctor or other health professional ever told you that you had emphysema?”; “Has a doctor or other health professional ever told you that you had chronic bronchitis?”* Patients with one or more of the following were considered to have a history of CVD: angina pectoris, coronary heart disease, myocardial infarction, congestive heart failure, or cerebrovascular disease.

### Measurements of mediator

Given that cigarette smoking is a major source of cadmium exposure, cadmium concentration was added as a mediator between smoking and mortality^35,36^. The NHANES website (https://www.cdc.gov/nchs/nhanes/Index.htm) provides details regarding the collection and processing of blood samples for the measurement of cadmium content. Inductively coupled plasma mass spectrometry (ICP-MS, ELAN® DRC II; PerkinElmer, Waltham, MA) was used at the CDC’s National Center for Environmental Health to measure the amount of cadmium in whole blood.

### Measurements of outcomes

Based on a probabilistic match between the NHANES and the National Death Index death certificates until December 31, 2019, mortality statistics for NHANES were retrieved from public-use linked mortality files at the National Center for Health Statistics.

### Statistical analysis

Continuous and categorical variables of demographic characteristics, underlying diseases, anthropometric index, and blood test results are presented using mean or frequency (%) according to smoking status, respectively. Independent t-tests and Pearson’s chi-squared tests were used to compare results. We also used sampling weights to account for multistage and stratified sampling. Multiple Cox regression analysis was utilized to assess the hazard ratio (HR) of smoking status with or without passive smoking and smoking pack years for all-cause and disease-specific mortality by adjusting for age, sex, race/ethnicity, alcohol consumption, and comorbidities at baseline survey, such as cancer classification, hypertension, diabetes, hyperlipidemia, and previous CVD events according to smoking status. The follow-up duration was calculated as the time from the first anthropometric and clinical measurements to death or last follow-up (December 31, 2019).

We performed propensity score matching (PSM) with age, sex, race/ethnicity, current smoking status, and smoking pack-years, considering the heterogeneity of demographic, clinical, and laboratory characteristics according to passive smoking status. We utilized 1:1 matching according to passive smoking status by the nearest neighbor method with a caliber of 0.25 using the R package “MatchIt”^37^. The Pearson correlation coefficient was used to investigate the correlation between (smoking pack years and cotinine) and cadmium concentrations before mediation analysis.

Using the R package “Regmedint”^38^, we performed regression-based causal mediation analysis to examine the direct influence of smoking status and the indirect effect via cadmium exposure. This R package is equivalent to the SAS mediation macro^39,40^. The total natural indirect effect (TNIE), pure natural indirect effect, total natural direct effect (TNDE), pure natural direct effect, and cumulative effect of smoking exposure and smoking status on mortality were calculated. R version 3.1.0 (R Foundation for Statistical Computing, Vienna, Austria; www.r-project.org) and the Statistical Package for the Social Sciences Statistics (version 24.0; IBM, Armonk, NY) were used for statistical analysis. Statistical significance was defined as *p* < 0.05.

### Ethics

All U.S. NHANES protocols were approved by the Research Ethics Review Board of the National Center for Health Statistics, U.S. Centers for Disease Control and Prevention (NCHS IRB/ERB Protocol Number: 1999–2004, Protocol #98-12; 2005–2010, Protocol #2005–06; 2011–2016, Protocol #2011–17) in accordance with the declaration of Helsinki. Informed consent was obtained from all participants.

## Results

### Baseline characteristics of the participants

A total of 52,730 (Model 1) and 34,781 participants (Model 2) from the 1999–2018 NHANES data were eligible for this study (Fig. 1). The demographical, clinical, and laboratory characteristics of the participants based on smoking status and presence or absence of passive smoking are described in Table 1 (Model 1) and Table 2 (Model 2).

**Table 1:** Baseline characteristics of primary analysis (Model1).

	Smoking status				Passive smoking status		
	Never-smoker	Former smoker	Current smoker	*p* value	Absence	Presence	*p* value
Unweighted (N)	30,089	11,647	11,001		43,913	8824	
Weighted (N)	116,400,000	45,360,000	45,160,000		172,800,000	34,070,000	
Age, years	45.1 ± 0.22	55.1 ± 0.27	42.2 ± 0.20	< 0.001	47.1 ± 0.20	44.4 ± 0.26	< 0.001
Female, sex, %	58.4 ± 0.3	42.8 ± 0.6	45.8 ± 0.6	< 0.001			< 0.001
Race/Ethnicity, %				< 0.001			< 0.001
Hispanic	16.0 ± 0.9	10.5 ± 0.7	11.3 ± 0.8		16 ± 0.9	10.5 ± 0.7	
Non-Hispnic White	63.2 ± 1.1	77.6 ± 0.9	69.6 ± 1.2		63.2 ± 1.1	77.6 ± 0.9	
Non-Hispnic Black	12.5 ± 0.6	6.9 ± 0.4	13.1 ± 0.7		12.5 ± 0.6	6.9 ± 0.4	
Other races	8.2 ± 0.4	5.0 ± 0.3	6.1 ± 0.4		8.2 ± 0.4	5 ± 0.3	
Smoking status, %							< 0.001
Never-smoker	NA	NA	NA		63.7 ± 0.5	18.6 ± 0.6	
Former smoker	NA	NA	NA		24.5 ± 0.4	9.0 ± 0.4	
Current smoker	NA	NA	NA		11.9 ± 0.2	72.4 ± 0.7	
Passive smoking, %	5.5 ± 0.2	6.8 ± 0.4	54.6 ± 1.1	< 0.001			
Smoking pack years	0	19.9 ± 0.35	19.2 ± 0.45	0.137	6.2 ± 0.15	20.4 ± 0.53	< 0.001
All cause death	8.4 ± 0.2	17.8 ± 0.6	12.8 ± 0.4	< 0.001	10.4 ± 0.3	16.4 ± 0.5	< 0.001
CVD related death	2.8 ± 0.1	5.1 ± 0.3	3.1 ± 0.2	< 0.001	3.2 ± 0.1	4.3 ± 0.3	< 0.001
Cancer related death	1.6 ± 0.1	4.5 ± 0.3	3.6 ± 0.2	< 0.001	2.3 ± 0.1	4.4 ± 0.3	< 0.001
Respiratory related death	0.4 ± 0	1.6 ± 0.1	1.5 ± 0.1	< 0.001	0.7 ± 0.1	1.7 ± 0.1	< 0.001

*CVD* cardiovascular disease.

**Table 2:** Baseline characteristics of secondary analysis (Model 2).

	Smoking status				Passive smoking status		
	Never-smoker	Former smoker	Current smoker	*p* value	Absence	Presence	*p* value
Unweighted (N)	19,440	8767	6574		29,576	5205	
Weighted (N)	83,240,000	37,250,000	28,680,000		128,000,000	21,130,000	
Age, years	47.9 ± 0.24	56.1 ± 0.30	44.9 ± 0.25	0.729	49.6 ± 0.17	47.7 ± 0.74	< 0.001
Female, sex, %	58.0 ± 0.4	42.6 ± 0.7	46.9 ± 0.8		52.3 ± 0.3	50.4 ± 0.8	0.025
Race/Ethnicity, %				< 0.001			< 0.001
Hispanic	13.9 ± 0.9	9.8 ± 0.7	10.0 ± 0.8		13.0 ± 0.8	7.0 ± 0.7	
Non-Hispnic White	67.6 ± 1.2	79.3 ± 1.0	71.7 ± 1.2		71.1 ± 1.0	72.3 ± 1.5	
Non-Hispnic Black	11.1 ± 0.6	6.2 ± 0.4	11.9 ± 0.7		9.0 ± 0.5	15.9 ± 1.0	
Other races	7.5 ± 0.4	4.7 ± 0.3	6.4 ± 0.4		6.9 ± 0.3	4.8 ± 0.5	
Smoking status, %							< 0.001
Never-smoker	NA	NA	NA		62.0 ± 0.5	18.2 ± 0.7	
Former smoker	NA	NA	NA		27.2 ± 0.4	11.4 ± 0.6	
Current smoker	NA	NA	NA		10.8 ± 0.3	70.4 ± 0.9	
Passive smoking, %	4.6 ± 0.2	6.5 ± 0.4	51.9 ± 1.2	< 0.001	NA	NA	
Smoking Pack Years	NA	20.5 ± 0.42	20.8 ± 0.58	0.687	6.9 ± 0.17	22.8 ± 0.73	< 0.001
Drinkers, %	62.9 ± 0.9	79.8 ± 0.7	82.5 ± 0.7	< 0.001	69.6 ± 0.7	78.4 ± 0.9	< 0.001
History of cancer, %	9.0 ± 0.3	16.2 ± 0.5	8.4 ± 0.5	< 0.001	10.8 ± 0.3	9.9 ± 0.6	< 0.001
Urogenital cancer	0.2 ± 0.1	0.7 ± 0.1	0.4 ± 0.1		0.3 ± 0.1	0.4 ± 0.1	
Prostate cancer	0.7 ± 0.1	2.3 ± 0.2	0.5 ± 0.1		1.1 ± 0.1	0.7 ± 0.1	
Uterine cervical cancer	0.5 ± 0.1	0.7 ± 0.1	1.6 ± 0.2		0.6 ± 0.1	1.7 ± 0.3	
Breast cancer	1.7 ± 0.1	1.9 ± 0.2	0.9 ± 0.2		1.7 ± 0.1	1.2 ± 0.2	
GI cancer	0.5 ± 0.1	1.0 ± 0.1	0.4 ± 0.1		0.6 ± 0.1	0.5 ± 0.1	
Respiratory cancer	0.1 ± 0	0.7 ± 0.1	0.2 ± 0.1		0.2 ± 0.1	0.3 ± 0.1	
Skin cancer	3.5 ± 0.2	6.3 ± 0.4	2.7 ± 0.3		4.3 ± 0.2	2.7 ± 0.3	
Other cancer	1.8 ± 0.1	2.7 ± 0.2	1.8 ± 0.2		2.0 ± 0.1	2.4 ± 0.3	
Previous CVD, %	6.9 ± 0.2	15.6 ± 0.5	11.1 ± 0.5	< 0.001	9.4 ± 0.2	13.2 ± 0.7	< 0.001
DM	13.1 ± 0.3	19.6 ± 0.6	11.6 ± 0.4	< 0.001	14.3 ± 0.3	14.7 ± 0.6	0.595
Hypertension, %	41.8 ± 0.6	56.8 ± 0.9	40.9 ± 0.9	< 0.001	45.2 ± 0.6	46.5 ± 1	0.170
Dyslipidemia, %	44.9 ± 0.6	59.3 ± 0.8	48 ± 0.8	< 0.001	48.9 ± 0.5	50.4 ± 0.9	0.125
COPD, n (%)	4.6 ± 0.2	10.2 ± 0.4	13.4 ± 0.6	< 0.001	6.5 ± 0.2	14.8 ± 0.7	< 0.001
BMI, kg/m^2^	29.2 ± 0.10	29.8 ± 0.11	28.3 ± 0.11	< 0.001	29.2 ± 0.08	29.0 ± 0.15	0.376
Systolic BP, mmHg	122.3 ± 0.21	125.7 ± 0.31	121.5 ± 0.32	0.459	123.0 ± 0.19	123.3 ± 0.37	0.328
Diastolic BP, mmHg	71.4 ± 0.17	70.9 ± 0.23	71.0 ± 0.24	0.022	71.2 ± 0.17	71.5 ± 0.28	0.237
Fasting glucose, mg/dL	105.5 ± 0.45	110.6 ± 0.60	105.3 ± 0.57	0.042	106.7 ± 0.38	107.0 ± 0.74	0.710
Hemoglobin A1c, %	5.6 ± 0.01	5.7 ± 0.02	5.6 ± 0.02	< 0.001	5.6 ± 0.01	5.7 ± 0.02	0.014
Total cholesterol, mg/dL	197.3 ± 0.50	199.2 ± 0.79	201.5 ± 0.85	< 0.001	198.0 ± 0.48	202.1 ± 0.97	< 0.001
Triglycerides, mg/dL	137.9 ± 1.60	153.1 ± 2.75	161.1 ± 2.76	< 0.001	143.9 ± 1.53	160.4 ± 3.31	< 0.001
HDL cholesterol, mg/dL	54.4 ± 0.19	53.3 ± 0.28	50.7 ± 0.29	< 0.001	53.8 ± 0.19	51.0 ± 0.34	< 0.001
Blood Cadmium, μg/L	0.30 ± 0.003	0.41 ± 0.005	1.18 ± 0.018	< 0.001	0.40 ± 0.004	1.07 ± 0.021	< 0.001
Cotinine, μg/L	9.8 ± 0.67	27.4 ± 1.88	220.0 ± 2.79	< 0.001	32.1 ± 1.01	191.9 ± 3.51	< 0.001
All cause death (%)	8.3 ± 0.3	16.6 ± 0.6	13.2 ± 0.5	< 0.001	10.4 ± 0.3	16.9 ± 0.6	< 0.001
CVD related death	2.9 ± 0.2	4.9 ± 0.3	3.2 ± 0.3	< 0.001	3.3 ± 0.1	4.6 ± 0.4	< 0.001
Cancer related death	1.6 ± 0.1	4.1 ± 0.3	3.9 ± 0.3	< 0.001	2.3 ± 0.1	4.6 ± 0.4	< 0.001
Respiratory related death	0.3 ± 0.1	1.6 ± 0.1	1.7 ± 0.2	< 0.001	0.7 ± 0.1	2.0 ± 0.2	< 0.001

*GI* gastrointestinal, *CVD* cardiovascular disease, *DM* diabetes mellitus, *COPD* chronic obstructive pulmonary disease, *BMI* body mass index, *BP* blood pressure, *HDL* high-density lipoprotein.

In model 1, more females were never-smokers (58.4%), whereas more males were smokers (former: 57.2%, current: 55.2%). Former smokers were older than current smoker (55.1 ± 0.27 years vs. 42.2 ± 0.20 years; *p* < 0.001). The number of smoking pack-years of current smokers and former smokers was not significantly different each other (19.2 ± 0.45 vs. 19.9 ± 0.35; *p* = 0.137). All-cause mortality and disease-specific mortality were the highest in former smokers.

The mean age of participants without passive smoking was higher than that of participants with passive smoking (47.1 ± 0.20 years vs. 44.4 ± 0.26 years; *p* < 0.001) in model 1. All-cause and disease-specific mortality of the participants with passive smoking were higher than those of the participants without passive smoking. Similar to model 1, more females were never-smokers (58.0%), and more males were smokers (former: 57.4%, current: 54.1%) in model 2. Besides, mean age of former smokers (56.1 ± 0.30 years) was also higher than that of current smokers (44.9 ± 0.25 years). Furthermore, the number of smoking pack-years of current smokers (20.8 ± 0.58) was not significantly different from that of former smokers (20.5 ± 0.42). Mortality and the prevalence of cancer and metabolic diseases such as CVD, DM, hypertension, dyslipidemia were the highest in former smokers, while the prevalence of COPD was the highest in current smoker.

The mean age of participants without passive smoking was higher than that of participants with passive smoking (49.6 ± 0.17 years vs. 47.7 ± 0.74 years; *p* < 0.001) in model 2. Mortality and the prevalence of COPD and metabolic disease such as CVD, DM, hypertension, dyslipidemia were higher in the participants with passive smoking, while the prevalence of cancer was higher in the participants without passive smoking.

### Effect of smoking and passive smoking on all-cause and disease-specific mortality

**Figure 2:**
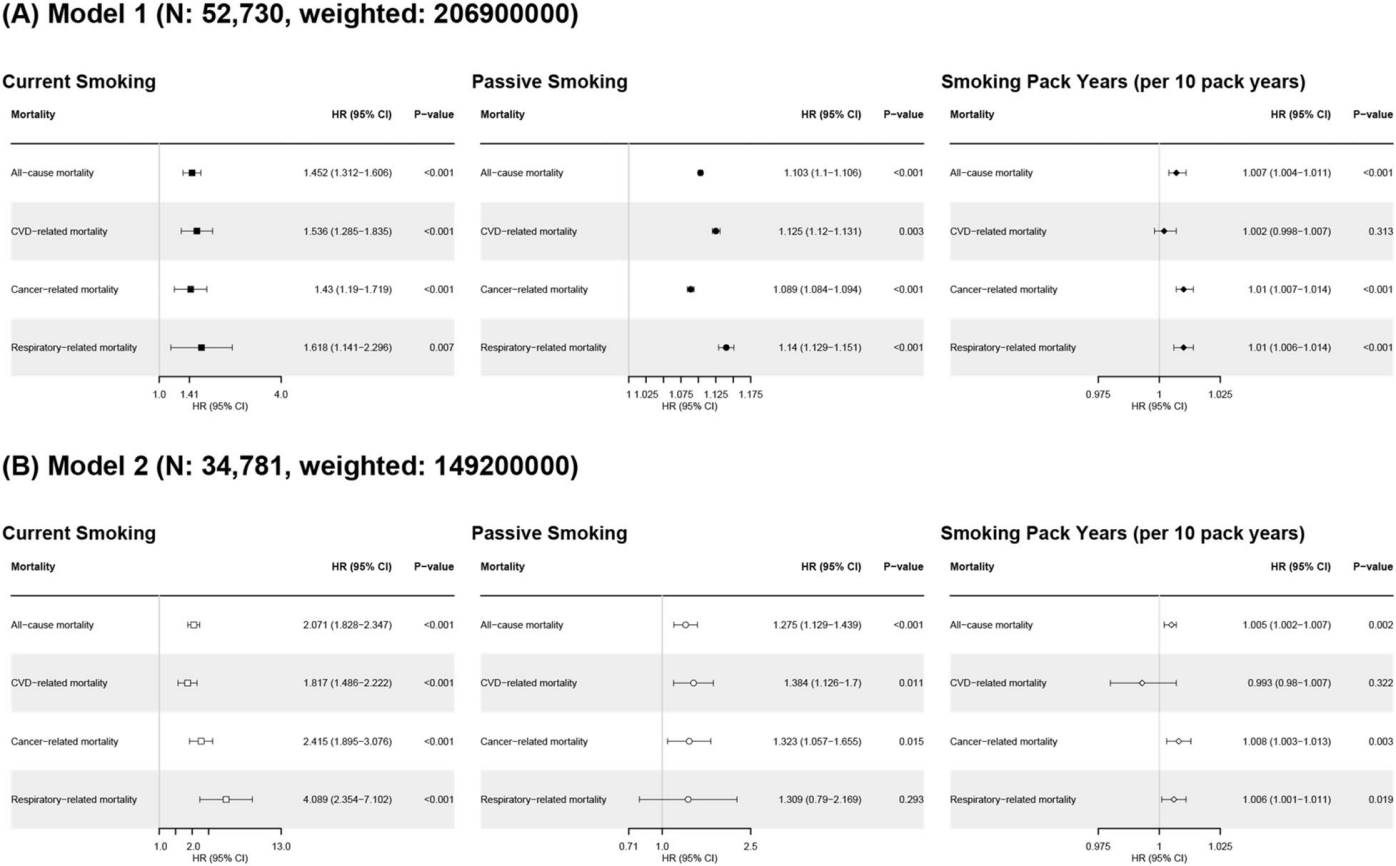
Hazard ratio for all-cause and disease-specific mortality according to smoking status and passive smoking. (**A**) Model 1 (**B**) Model 2. Model 1: Age, sex, and races/ethnicities. Model 2: Model 1 + alcohol consumption status, body mass index, cancer type, chronic obstructive pulmonary disease, cardiovascular disease, diabetes, hypertension, and dyslipidemia. *HR* hazard ratio, *CI* confidence interval.

**Figure 3:**
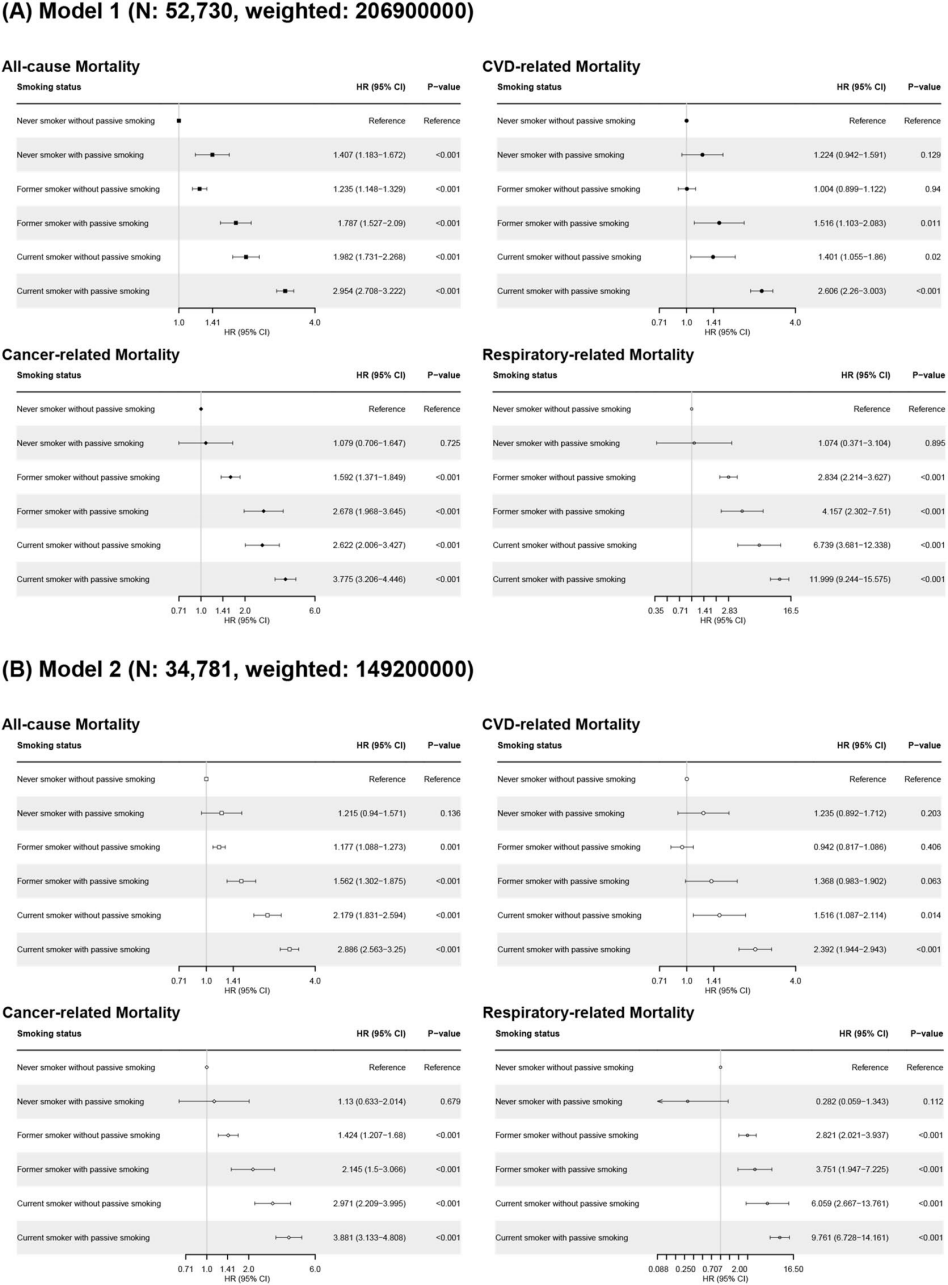
Hazard ratio for all-cause and disease-specific mortality in subgroup according to smoking and passive smoking status. Model 1: Age, sex, and races/ethnicities. Model 2: Model 1 + alcohol consumption status, body mass index, cancer type, chronic obstructive pulmonary disease, cardiovascular disease, diabetes, hypertension, and dyslipidemia. *HR* hazard ratio, *CI* confidence interval.

**Figure 4:**
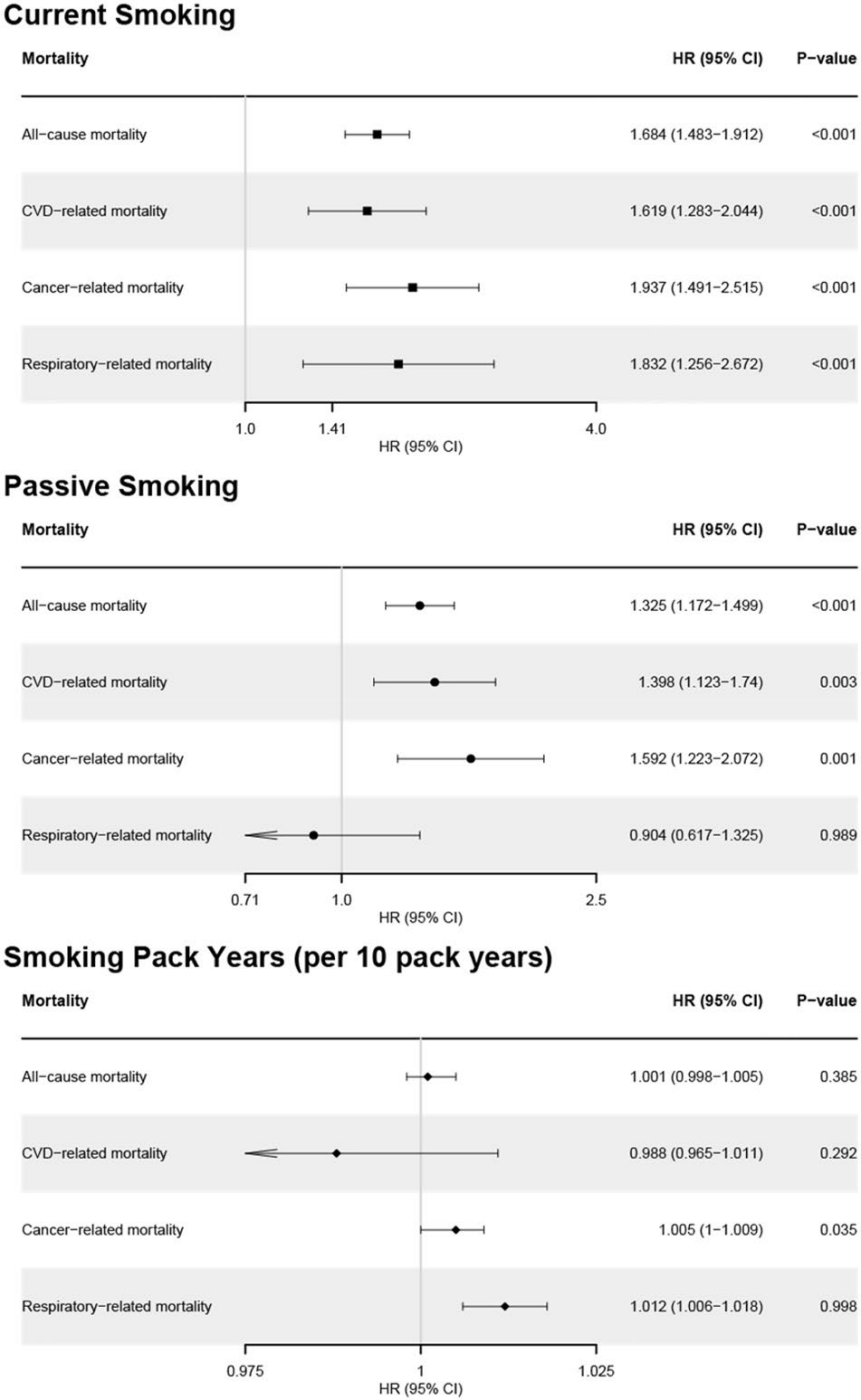
Hazard ratio for all-cause and disease-specific mortality according to passive smoking in propensity matching group according to passive smoking. Propensity matching with age, sex, races/ethnicities, alcohol consumption status, body mass index, cancer history, chronic obstructive pulmonary disease, cardiovascular disease, diabetes, hypertension, and dyslipidemia.

In primary analysis, current smoking and passive smoking were related to increased all-cause mortality (current smoker compared to never-smoker, HR 1.452, 95% confidence intervals [CIs] 1.312–1.606, *p* < 0.001; participants with passive smoking compared to participants without passive smoking, HR 1.103, 95% CIs 1.1–1.106, *p* < 0.001), CVD-related mortality (current smoking, HR 1.536, 95% CIs 1.285–1.835, *p* < 0.001; passive smoking, HR 1.125, 95% CIs 1.12–1.131, *p* < 0.001), cancer-related mortality (current smoking, HR 1.43, 95% CIs 1.19–1.719, *p* < 0.001; passive smoking, HR 1.089, 95% CIs 1.084–1.094, *p* < 0.001), and respiratory related mortality (current smoking, HR 1.618, 95% CIs 1.141–2.296, *p* = 0.007; passive smoking, HR 1.14, 95% CIs 1.129–1.151, *p* < 0.001). Smoking pack years was also related to increased all-cause, cancer-related, and respiratory-related mortality, but not related to CVD-related mortality (Fig. 2A). Adjusting for comorbidity, the HRs for all-cause and disease-specific mortality in relation to passive smoking are presented in Fig. 2B. Passive smoking increased the risk of all-cause (HR 1.275, 95% CIs 1.129–1.439, *p* < 0.001), CVD-related (HR 1.384, 95% CIs 1.126–1.7, *p* = 0.011), and cancer related deaths (HR 1.323, 95% CIs 1.057–1.655, *p* = 0.015). The all-cause mortality and disease-specific mortality of subgroups classified by smoking and passive smoking status are shown in Fig. 3. Passive smoking showed a synergistic effect with smoking status on the risk of all-cause mortality. In particular, current smokers with passive smoking had the highest risk of all-cause and disease-specific deaths. Additional analysis with PSM data according to passive smoking showed that passive smoking increased the risk of all-cause and disease specific deaths (All-cause death, HR 1.325, 95% CIs 1.172–1.499, *p* < 0.001; CVD-related death, HR 1.398, 95% CIs 1.123–1.74, *p* = 0.003; cancer-related death, HR 1.592, 95% CIs 1.223–2.072, *p* < 0.001; Fig. 4).

### Mediation effect of cadmium between smoking and mortality

We performed a correlation analysis of cotinine and smoking pack-years with blood cadmium concentrations (Table 3). Cotinine and smoking pack-years showed a positive correlation with cadmium in every subgroup according to smoking status and passive smoking.

**Table 3:** Correlation between smoking and cadmium.

	Total		Without passive smoking		With passive smoking	
	Pearson correlation coefficient	*p* value	Pearson correlation coefficient	*p* value	Pearson correlation coefficient	*p* value
Total						
Cotinine, ng/mL	0.528	< 0.001	0.403	< 0.001	0.425	< 0.001
Smoking pack years	0.184	< 0.001	0.126	< 0.001	0.115	< 0.001
Current smoker						
Cotinine, ng/mL	0.328	< 0.001	0.376	< 0.001	0.244	< 0.001
Smoking pack years	0.109	< 0.001	0.114	< 0.001	0.056	< 0.001
Former smoker						
Cotinine, ng/mL	0.131	< 0.001	0.09	< 0.001	0.286	< 0.001
Smoking pack years	0.036	< 0.001	0.024	< 0.001	0.21	< 0.001
Never smoker						
Cotinine, ng/mL	0.09	< 0.001	0.063	< 0.001	0.241	< 0.001

**Figure 5:**
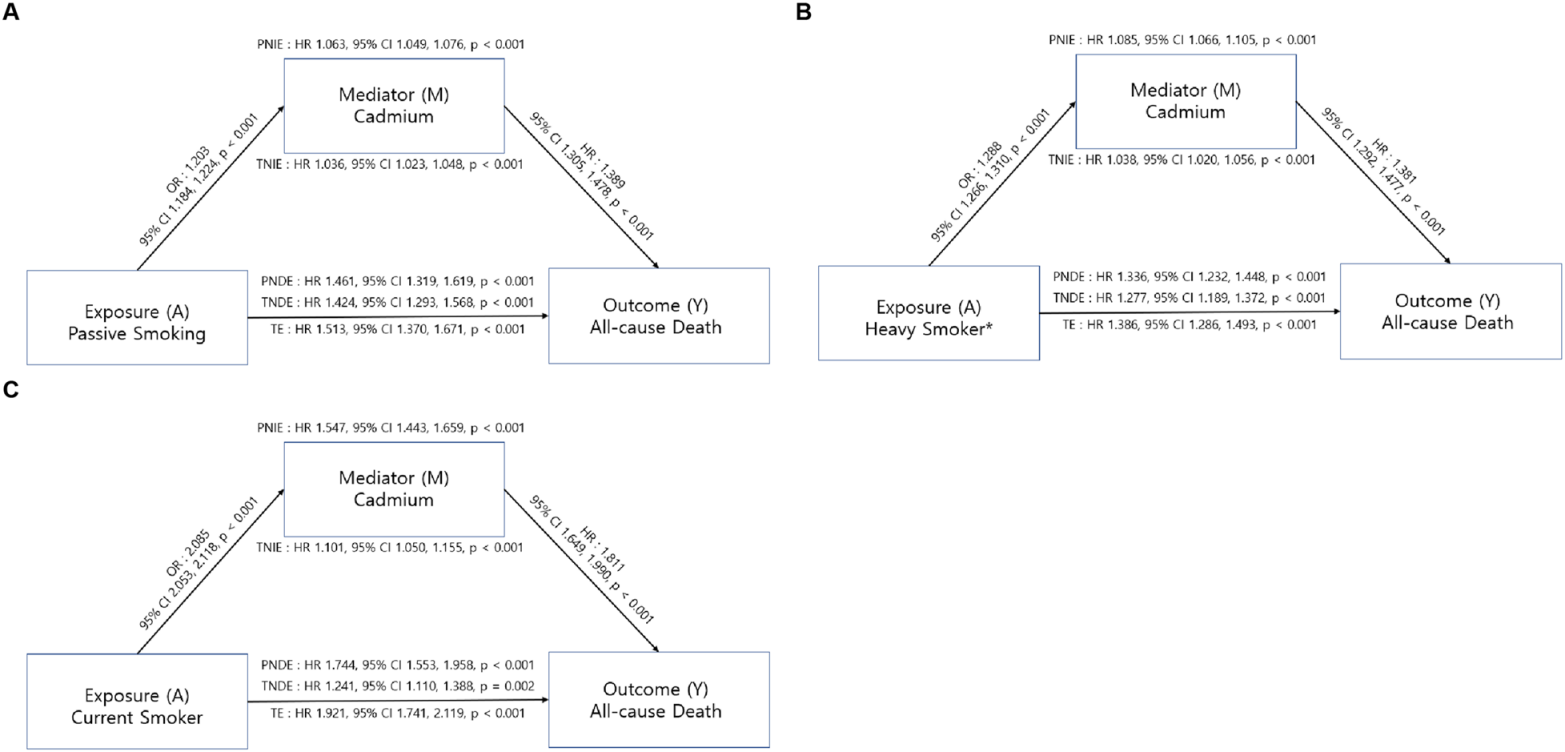
Mediation analysis for all-cause mortality. (**A**) The effect of passive smoking on all-cause mortality (**B**) The effect of heavy smoker on all-cause mortality (**C**) The effect of current smoking on all-cause mortality. Values were adjusted for age, sex, races/ethnicities, alcohol consumption status, smoking status, smoking pack years, passive smoking stutus, BMI, cancer type, COPD, CVD, diabetes, hypertension, and dyslipidemia. *BMI* body mass index, *COPD* chronic obstructive pulmonary disease, *CVD* cardiovascular disease, *HR* hazard ratio, *CI* confidence interval. The effect of heavy smoker on all-cause mortality. *PNIE* pure natural indirect effect, *TNIE* total natural indirect effect, *PNDE* pure natural direct effect, *TNDE* total natural direct effect, *TE* total effect, *HR* hazard ratio, *CI* confidence interval. *Heavy smokers were defined as participants whose smoking pack years were more than 75 percentile among ever-smokers.

Figure 5 shows the results of the mediation analysis to test the effect of smoking and passive smoking on all-cause mortality mediated by the blood cadmium concentration. The total effect of current smoking increased the risk of all-cause mortality (HR 1.921, 95% CI 1.741–2.119, *p* < 0.001). Current smoking was associated with an increase in blood cadmium concentration (OR 2.085, 95% CI 2.053–2.118, *p* < 0.001) and an increased risk of mortality through cadmium (TNIE: HR 1.101, 95% CI 1.050–1.115, *p* < 0.001). Furthermore, current smoking also increased the risk of all-cause death, directly (TNDE: HR 1.241, 95% CI 1.110–1.388, *p* = 0.001). Similar to current smoking, passive smoking and history of heavy smoking, defined as more than the 75th percentile of smoking pack years among current/former smokers, showed a significant direct and indirect effect mediated by cadmium on all-cause mortality (passive smoking TNIE: HR 1.036, 95% CI 1.023–1.048, *p* < 0.001, TNDE: HR 1.424, 95% CI 1.293–1.568, *p* < 0.001; heavy smoking TNIE: HR 1.038, 95% CI 1.020–1.056, *p* < 0.001, TNDE: HR 1.277, 95% CI 1.189–1.372, *p* < 0.001; Fig. 5).

## Discussion

To the best of our knowledge, this study is one of the largest in scale to widely investigate the impact of smoking on all-cause and disease-specific mortality (i.e., CVD, COPD, and cancer mortality). Moreover, this study firstly identified the mediation effect of cadmium between smoking and mortality. A number of previous studies reported the harmful effect of cigarette smoking and passive smoking on mortality^41–43^. This study revealed consistent results with previous ones, that current smoking, smoking pack-years, and passive smoking increased both all-cause and disease-specific mortality. Importantly, this study found synergistic effect of passive smoking among former or current smokers, of which little has been known.

There are two types of Tobacco smoke: mainstream smoke (the smoke that a smoker inhales and exhales) and sidestream smoke (released by cigarette). It contains more than 7000 different chemicals. Passive smoking is mainly composed of sidestream smoke and contains more than 50 carcinogens^44^. Although mainstream and sidestream smoke have similar composition there are some compounds which concentration is particularly high in passive smoking^45^. In vivo toxicity experiments have shown that sidestream smoke is more toxic and carcinogenic than mainstream smoke. In particular, respiratory epithelial tissues have been identified to be more susceptible to the gas/vapor phase of smoke^46,47^. Therefore, passive smoking can cause chronic epithelial inflammation and DNA damage and increase the risk of lung cancer^48^.

Cadmium is heavy metal which is biologically non-essential and with harmful effects on the human body^49^. The International Agency for Research on Cancer (IARC) categorized cadmium as “carcinogenic to humans (Group 1)” due to epidemiological research linking it to an increased risk of lung, kidney, and prostate cancers^50^. Through inhalation and consumption of tainted food and water, humans are exposed to Cd. Another significant exposure route of Cd is smoking. The amount of Cd in a cigarette is approximately 1–2 ug^26^. This study also found that the cotinine levels were positively correlated with cadmium levels in both smokers and non-smokers. Especially, even for non-smoker, cotinine level through passive smoking significantly increases the level of cadmium. These results provided epidemiologic evidence of cigarette smoking and passive smoking as the major source of cadmium exposure in humans. Furthermore, the present study revealed that cigarette smoking and passive smoking had both direct effect and indirect effect through cadmium by mediation analysis. Our results suggest that cadmium from cigarette might have played an important role as pathogenesis of smoking-related diseases such as CVD, COPD, and cancer.

This research was conducted using a large dataset in the United States, which allows advantageous data on mortality, smoking status, passive smoking, and disease status for all included participants. In addition, the influence of covariates on the analysis results was investigated by dividing the model according to the presence or absence of covariates. Furthermore, propensity score matching was performed to minimize the influence of the covariates on the analysis results. However, our study has several limitations. First, the effect of exposure level of passive smoking on all-cause and disease-specific mortality was not evaluated. Second, the effects of covariates which can affect cadmium concentration of the human body (industrial activity, food, etc.) in addition to cigarette smoke were not evaluated. Despite these limitations, we showed the role of passive smoking on mortality (all-cause and disease-specific mortality) in both of smokers and non-smokers. Moreover, we also presented a causal relationship between smoking, cadmium, and mortality.

## Conclusion

In conclusion, we demonstrated the role of smoking status and passive smoking on all-cause mortality and disease-specific mortality using large-scale data from the NHANES. Notably, passive smoking showed a synergistic harmful effect with smoking status on the risk of mortality. In particular, current smokers with passive smoking had the highest risk of all-cause and disease-specific deaths. In addition, the accumulation of cadmium in the blood due to smoking and passive smoking mediates the increased risk of all-cause mortality. Further studies are needed to monitor and treat cadmium toxicity to improve smoking-related mortality rates.

## Data Availability

The dataset supporting the conclusions of this article is available in the CDC repository. [National Health and Nutrition Examination Survey in https://www.cdc.gov/nchs/nhanes/index.htm].
